# Effects of Substitution of Corn with Ground Brown Rice on Growth Performance, Nutrient Digestibility, and Gut Microbiota of Growing-Finishing Pigs

**DOI:** 10.3390/ani11020375

**Published:** 2021-02-02

**Authors:** Sheena Kim, Jin Ho Cho, Younghoon Kim, Hyeun Bum Kim, Minho Song

**Affiliations:** 1 Department of Animal Resources Science, Dankook University, Cheonan 31116, Korea; sheen915@gmail.com; 2 Division of Animal and Dairy Science, Chungnam National University, Daejeon 34134, Korea; 3 Division of Food and Animal Science, Chungbuk National University, Cheongju 28644, Korea; jinhcho@chungbuk.ac.kr; 4 Department of Agricultural Biotechnology and Research Institute of Agriculture and Life Sciences, Seoul National University, Seoul 08826, Korea; ykeys2584@snu.ac.kr

**Keywords:** apparent total tract digestibility, brown rice, carcass characteristics, growing-finishing pigs, growth performance, gut microbiota

## Abstract

**Simple Summary:**

Corn is the main feed ingredient used in swine diets as an energy source due to its abundant production and nutrient contents. In South Korea, most of the corn for animal diets depends on import from other countries—more than 7.5 million tons per year. Thus, there is a need to find alternative ingredients to substitute corn in pig diets. Although there are variations depending on the degree of milling, brown rice has similar or better nutrient contents compared to corn. In addition, it is known to have excellent digestibility due to its smaller starch structure and granule size and less non-starch polysaccharides and anti-nutritional factors than corn. As a result of evaluating the effects of replacing corn with brown rice in pig diets, changes in gut microbiota were observed when corn was replaced with brown rice for a long time, but there were no differences on growth performance and carcass characteristics. Therefore, it has been confirmed that brown rice can replace corn in swine diets and the use of brown rice as a pig feed ingredient may be the basis for increasing feed self-sufficiency and enabling a stable feed supply.

**Abstract:**

The present study was conducted to evaluate the effects of replacing corn with brown rice on growth performance, nutrient digestibility, carcass characteristics, and gut microbiota of growing and finishing pigs. A total of 100 growing pigs (23.80 ± 2.96 kg BW; 10 weeks of age) were randomly allotted to 4 dietary treatments (5 pigs/pen; 5 replicates/treatment) in a randomized complete block design (block = BW) as follows: corn-soybean meal basal diet (CON) and replacing corn with 50% (GBR50), 75% (GBR75), and 100% (GBR100) of ground brown rice. Each trial phase was for 6 weeks. During the growing period, there were no differences on growth performance and nutrient digestibility among dietary treatments. Similarly, no differences were found on growth performance, nutrient digestibility, and carcass characteristics of pigs during the finishing period among dietary treatments. As a result of the beta diversity analysis, microbial populations were not clustered between CON and GBR100 during the growing phase, but clustered into two distinct groups of CON and GBR100 during the finishing phase. In conclusion, brown rice can be added to the diets of growing-finishing pigs by replacing corn up to 100% without negatively affecting growth performance of the pigs; additionally, this may have an effect on changes in pig intestinal microbiota if continued for a long time.

## 1. Introduction

Corn is the main feed ingredient used in swine diets as an energy source due to its abundant production, nutrient contents, and relatively long storage. Rice is the world’s third most grown staple crop providing 50 percent of the world’s calories along with corn and wheat [1]. Since rice has long been a staple food for people and is more expensive than other grains, the use of rice as an animal feed ingredient has been restricted [2,3,4]. With the development of agricultural technology, rice production in Asia has gradually increased. Therefore, there have been many attempts to use rice and their by-products as animal feed ingredients.

Although the quality of nutrient contents varies depending on the degree of milling, except for whole grains (paddy rice), rice contains more starch than corn [5] and its structure and granule size are easier to digest than corn [6,7]. In addition, the fact that rice has fewer non-starch polysaccharides and anti-nutritional factors is effective in improving digestibility [8,9] and various polyphenols in rice can help modulation of immune responses [10], which can be beneficial to the gastrointestinal environment and health of pig.

The mammalian gut intestinal tract (GIT) has diverse and active microbial communities that provide important signals for the development of the immune system and for functions such as digesting and absorbing nutrients [11]. Diet composition affects metabolic activity by providing substrates that can be used by intestinal microorganisms. It can induce changes in microbial composition in various parts of the GIT and affect the health of the GIT [12]. Compared with corn, brown rice has less fiber contents and similar concentrations in gross energy (GE), crude protein (CP), and ether extracts, but the amount of starch, essential amino acids, and fatty acids in brown rice are relatively higher than those in corn [2,3,13].

Several studies indicate that brown rice can completely replace corn in swine diets for weanling and growing pigs without negative impacts on growth performance and apparent digestibility of nutrients [14,15,16]. However, there is limited research reported on impacts of different replacement rates of brown rice on growth performance and nutrient digestibility of growing-finishing pigs. Therefore, the objective of this experiment was to evaluate the effects of substitution of corn with brown rice at different levels on growth performance, nutrient digestibility, carcass characteristics, and gut microbiota in growing-finishing pigs.

## 2. Materials and Methods

The protocol of this experiment was reviewed and approved by Institutional Animal Care and Use Committee at the Chungnam National University, Daejeon, South Korea (approval #CNU-00780). This experiment was conducted at the Animal Research Center of Chungnam National University, Cheongyang, South Korea.

### 2.1. Experimental Design, Animals, and Diets

A total of 100 growing pigs [(Landrace × Yorkshire) × Duroc; 23.80 ± 2.96 kg of average initial body weight (BW); 10 weeks of age] were used in this experiment. These pigs were randomly allotted to 4 dietary treatments (5 pigs/pen; 5 replicates/treatment) in a randomized complete block design (block = BW). After finishing the study for growing period, pigs were randomly relocated within groups and then the study for finishing period was conducted. Dietary treatments were growing and finishing diets based on corn-soybean meal basal diet (CON) and three additional diets formulated by replacing corn with 50% (GBR50), 75% (GBR75), and 100% (GBR100) of ground brown rice. The brown rice used in this experiment was ground with an average particle size of 0.5 mm (0.4–0.6 mm). The pigs were fed respective dietary treatments for 6 and 6 weeks for the growing and finishing periods, respectively. The dietary treatments were formulated to meet or exceed the nutrient requirement of growing and finishing pigs [17] and had similar CP, calcium, and phosphorus except metabolizable energy (ME). All diets were fed as mash form and did not include animal plasma, antibiotics, zinc oxide, or any additives to avoid their physiological or antibacterial effects. The environmental conditions were automatically controlled by a mechanical system with the ambient temperature maintained at 18–21 °C and the lighting program regulated on a 12-h light/dark cycle. Pigs had ad libitum access to feed and water throughout the entire experimental period, except for one day before slaughter.

### 2.2. Sample Collection

Pigs were individually weighed at 1 (the first day), 42, and 84 d to measure average daily gain (ADG). Feed intake was recorded for each pen to calculate average daily feed intake (ADFI) and feed efficiency (G:F) for growth performance of growing and finishing periods. At the beginning of the last week of each period, 0.2% chromic oxide as an indigestible marker was mixed into each dietary treatment and the diets were fed to pigs during the last week of each period. For the periods, initial 4 days were adaptation days and feces were collected with anal massage method for 3 consecutive days. The fecal samples were collected from randomly selected 1 pig in each pen and stored at −80 °C for analysis to determine apparent total tract digestibility (ATTD) of nutrients. Three pigs from CON and GBR100 were randomly selected to collect feces on the last day of each experimental phase (d 42 and 84) and stored at −80 °C for analysis to verify their microbiota changes by pyrosequencing analysis. 

### 2.3. Slaughter and Carcass Evaluation

At the end of the experiment, the pigs were transferred to a local commercial slaughterhouse and treated with conventional process. The live BW of pig in each treatment was recorded just before slaughter. The hot carcass weight (HCW) was recorded and dressing percentage was calculated by comparing final BW and HCW. Then, carcasses were chilled at 2 °C for 24 h. At 24 h post-mortem, the right-side loin was taken between the 10th and 11th ribs to measure back fat thickness according to National Pork Producers Council [18].

### 2.4. Chemical Analysis

Diet and frozen fecal samples were oven-dried at 135 °C for 2 h and then finely ground before chemical analysis. Prepared diets and fecal samples were analyzed for dry matter (DM; method 930.15) [19], nitrogen by kjeldahl method (method 988.05) [19], GE using a bomb calorimeter (Parr 1281 Bomb Calorimeter, Parr Instrument Co., Moline, IL, USA), and chromium concentration using an absorption spectrophotometer (Hitachi Z-5000 Absorption Spectrophotometer, Hitachi High-Technologies Co., Tokyo, Japan) [20]. The ATTD of DM, GE, and CP were calculated for each sample according to Stein et al. [21].

### 2.5. 16S rRNA Gene Sequencing and Analysis of Fecal Microbiota

Total DNA representing the fecal microbiota was extracted from 300 mg of fecal contents per sample using QIAamp DNA Stool Mini Kit (Qiagen, Hilden, Germany) following manufacturer’s instructions. DNA concentration and quality were assessed using NanoDrop ND-1000 spectrophotometer (NanoDrop Technologies, Wilmington, DE, USA). Genomic DNA was stored at −70 °C until further analysis.

The V4 region of the 16S rRNA genes was amplified by PCR using featured primers as listed previously [22]. The 16S rRNA gene amplicons were sequenced using the Illumina MiSeq platform according to the manufacturer’s instructions. All sequencing was performed at Macrogen Inc., Seoul, South Korea. 

Raw sequence data were quality filtered using the Mothur software to remove low-quality sequences [23]. Sequences that are less than 100 bp in length or containing ambiguous sequences were eliminated [24] and the chimeric sequences were further removed using the UCHIME algorithm implemented in Mothur software to minimize the effect of random sequencing errors [25]. The remaining high quality sequences were classified into operational taxonomic units (OTUs) with an OTU definition at a similarity cutoff of 97% [26]. Taxonomic assignment and microbial alpha diversity analysis were conducted using QIIME. The sequence number was normalized by the random selection of the same sequence per sample to conduct downstream analyses. Then, microbial alpha diversities, such as observed OTUs, Chao1, Shannon, and Simpson, were measured to compare the microbial diversities between groups. In addition, the differences of microbial communities among groups were compared by the beta diversity (principal coordinates analysis, PCoA) based on the weighted UniFrac distance of fecal microbiota in pigs.

### 2.6. Statistical Analysis

Data were analyzed using the PROC MIXED of SAS (SAS Inst. Inc., Cary, NC, USA) in a randomized complete block design (block = BW). Experimental unit was the pen. The statistical model for growth performance, ATTD of nutrients, and carcass characteristics of pigs included effect of dietary treatments as a fixed effect and initial BW as a random effect. Pair-wise comparisons were performed among dietary treatments when main effects of dietary treatments were observed. Results are expressed as mean ± standard error of mean (SEM). Statistical significance and tendency were considered at *p* < 0.05 and 0.05 ≤ *p* < 0.10, respectively.

## 3. Results

### 3.1. Growth Performance, Nutrient Digestibility, and Carcass Characteristics

The dietary treatments were formulated to have similar concentrations of CP and GE. The analyzed concentrations of CP and GE in the diets were 19.43–20.64% and 4325–4446 kcal/kg, respectively, for growing pigs and 16.78–18.54% and 4275–4486 kcal/kg, respectively, for finishing pigs (Table 1).

There were no differences on ADG, ADFI, and G:F of pigs during growing phase among the dietary treatments (Table 2). Similarly, no treatment effects were observed on growth performance including ADG, ADFI and G:F of pigs during finishing phase (Table 2).

No differences were observed on ATTD of DM, energy, and CP for growing and finishing pigs among dietary treatments (Table 3). 

There were no differences on carcass characteristics such as live BW, HCW, dressing percentage, and back fat thickness among dietary treatments (Table 4).

### 3.2. Diversity of Gut Microbiota

After quality filtering, the average number of sequence reads obtained from growing pigs were 181,852 ± 19,196 (mean ± SD) for CON and 186,395 ± 16,116 for GRB100 (Table 5). The mean number of sequence reads generated from finishing pigs were 217,143 ± 73,819 for CON and 203,195 ± 46,636 for GBR100 (Table 5). Our data indicates that the alpha diversity indices were not significantly different between CON and GRB100 groups for both growing and finishing pigs (Table 5).

**Figure 1:**
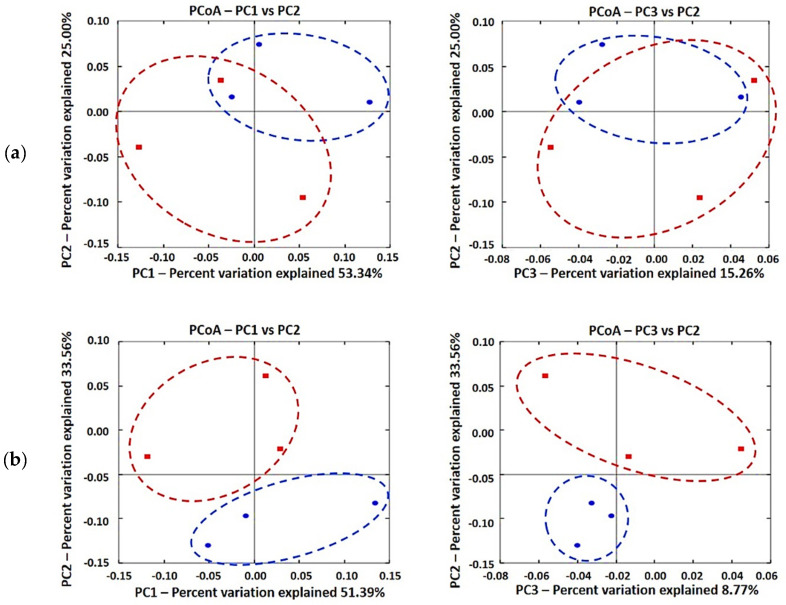
Principal coordinate analysis (PCoA) based on the weighted UniFrac distance of fecal microbiota in pigs: (**a**) growing pigs (**b**) finishing pigs. The 6 differentially abundant bacterial genera represent the number of variables in the model. Individual pig samples for treatments are designated with the following symbols: CON (red, square) and GBR100 (blue, circle).

The PCoA plot visually confirmed a distinct separation of microbial communities and characterized the differences of gut microbial communities between CON and GBR100 groups for growth phases (Figure 1). Microbial populations were not clustered based on diets during growing phase (Figure 1A), but they were clustered into two distinct groups of CON and GBR100 during finishing phase (Figure 1B).

**Figure 2:**
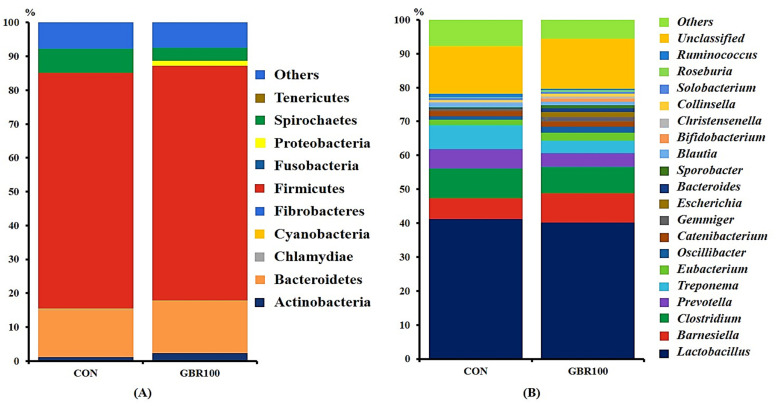
Stacked bar plots of the relative abundance of gut microbial communities at the phylum (**A**) and genus (**B**) levels in growing pigs fed the control diet (CON) and the diet with ground brown rice (GBR100) at week 6. The relative abundance of predominant bacterial taxa was averaged across all pigs between groups.

**Figure 3:**
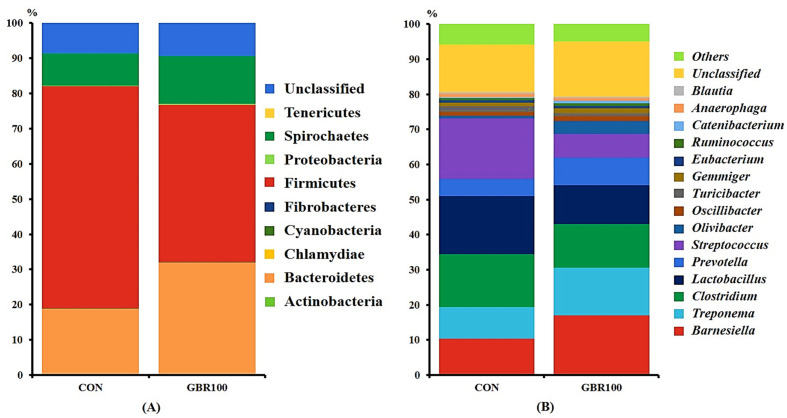
Stacked bar plots of the relative abundance of gut microbial community at the phylum (**A**) and genus (**B**) levels in finishing pigs fed the control diet (CON) and the diet with ground brown rice (GBR100) at week 12. The relative abundance of predominant bacterial taxa was averaged across all pigs between groups.

Taxonomic classification of the bacterial 16S rRNA genes at phylum and genus levels are shown in Figure 2 and Figure 3. A total of 12 phyla and 84 genera were identified in fecal samples of growing pigs, while 11 phyla and 88 genera represent fecal bacterial communities of finishing pigs.

During growing period, no significant differences of fecal microbial compositions at phylum level were detected between dietary treatment groups (Figure 2). Regardless of dietary treatments, Firmicutes, Bacteriodites, and Spirochaetes were dominant in both treatment groups. These three phyla accounted for approximately 90% of the total sequence reads for pigs in both treatment groups (Figure 2A). Likewise, there were no significant differences of the bacterial communities at the genus level between dietary treatment groups during growing period. Regardless of dietary treatments, *Lactobacillus* was the most abundant genus accounting for more than 40% of the total sequences for pigs in both treatment groups (Figure 2B).

However, the fecal bacterial compositions at phylum and genus levels were different between treatment groups during finishing period (Figure 3). The relative abundance of phylum Bacteroidetes was significantly higher (*p* < 0.05) in pigs fed GBR100 (31.80%) compared to that in pigs fed CON (18.70%). On the other hand, the relative abundance of phylum Firmicutes was significantly higher (*p* < 0.05) in CON pigs (63.11%) compared to that (44.66%) in GBR100 pigs (Figure 3A).

At genus level, the relative abundance of genus *Barnesiella* was relatively higher (*p* < 0.05) in GBR group (16.96%) compared to that in CON group (10.83%; Figure 3B). Meanwhile, genera *Lactobacillus* (10.95% vs. 16.53%) and *Streptococcus* (6.79% vs. 17.32%) were less abundant (*p* < 0.05) in finishing pigs fed GBR100 compared with those in finishing pigs fed CON (Figure 3B).

## 4. Discussion

As a result of analyzing the composition of diets, the GBR50 and GBR75 contained similar DM, CP, and energy to those of CON, but those of GBR100 was slightly lower than those of CON. It may be related to nutrient losses during grinding process of rice. Liu et al. [27] found that grinding method and time can change the composition of nutrients of rice such as amino acids and minerals. In general, rice is known to have the potential as a pig feed ingredient because its nutrient values are quite similar to corn and to be particularly characterized by high starch and low fiber contents. These characteristics make rice easier for pigs to digest and may contribute to modification of gut microbiota by providing less substrates for bacterial fermentation in the gut.

The results for growth performance in this experiment are in agreement with previous studies [15,28,29], which demonstrated no significant difference in overall growth performance when corn was completely or partially replaced with brown rice. On the other hand, some previous studies showed that pigs fed diets by completely or partially replacing corn with brown rice had significantly higher growth performance at various growth phases than those fed control diets without brown rice [14,30]. It has been mentioned that some farmers are reluctant to use brown rice as an animal feed ingredient due to its poor palatability [31]. However, the feed intake of pigs was not different between diets with and without brown rice in previous and present studies and thus the issue may be no longer considered as a factor for brown rice that may not be an animal feed ingredient. As the present study showed corn could be replaced up to 100% with brown rice, He et al. [32] also reported that more than 50% of corn could be replaced with brown rice because pigs fed diets with brown rice had higher feed conversion ratio than pigs fed control diets without brown rice. 

Brown rice is known to have excellent digestibility [15,33,34] because it has smaller starch structure and granule size and fewer non-starch polysaccharides and anti-nutritional factors than corn [35,36,37]. Li et al. [14] showed that the standard ileal digestibility and ATTD of amino acids as well as energy balance of brown rice in pig diets were superior to those of corn. Casas et al. [38,39] also found that brown rice had significantly better digestible energy, ME, and ATTD of Ca in growing pigs than corn. Moreover, Zhang et al. [15] reported that the digestibility of DM and GE of pigs fed diets with brown rice was slightly higher than those of pigs fed control diets without brown rice due to lower fiber contents of brown rice (1.87%) than corn (2.86%). The relationship between digestibility and fiber contents has been studied extensively, since fibers play a protective role between enzymes and substrates [40]. In this case, the fibers form a physical barrier to limit amylase access to starch granules to inhibit starch hydrolysis. However, Piao et al. [41] showed that there were no differences on nutrient digestibility of pigs between corn and brown rice. The results were consistent with the results of present study. Based on the previous and present results, brown rice can be a good energy source in pig diets [14] and an alternative to replace corn up to 100% with brown rice in pig diets [41]. 

Previous studies showed pig diets with brown rice did not change carcass characteristics of pigs compared with those without brown rice [42,43], but showed some changes in the composition of pork. The results for carcass characteristics were consistent with the results in present study. Further research is needed to investigate why those results were happened.

Recently, as it is known that the gut microbiota plays an important role in the health and disease of host [44,45,46,47,48,49,50,51], there were several previous studies about the intestinal microbiota related to growth performance of pigs [52,53,54,55,56]. For these changes, feed is one of the various factors affecting microbial composition [55,57,58]. From those points of view, Quan et al. [56] reported that pigs with high G:F had slightly higher microbial richness and evenness than pigs with low G:F. However, the present study showed no differences were found on G:F and microbial diversity between CON and GBR100 during the entire experimental period. On the other hand, there is a case of studying about the relationship between nutrient digestibility and microbiota and it showed that the microbiota was more changeable in a diet with low fiber than in a diet with high fiber [59]. However, there was limited information about the relationships and thus more research is needed.

Yang et al. [60] previously reported that the gut microbial shifts affected meat quality and took a crucial role as the major contributor to adiposity in pigs. Several studies reported the relationships between microbial compositions and carcass characteristics and showed higher abundance of *Lactobacillus*, *Oscilibacter*, *Roseburia*, and *Clostridium* in pigs with high-quality pork compared to those in pigs with low-quality pork [61,62]. Park et al. [61] showed that the relative abundance of those four genera was 5% higher in pigs with high-quality pork compared to those in pigs with low-quality pork. However, their relative abundances were lower in this experiment compared to the previous results reported by Park et al. [61] and the difference was less than 1% between treatment groups (*Oscilibacter* 0.98% vs. 1.31%, *Roseburia* 0.87% vs. 0.05% for CON and GBR100, respectively). The *Oscilibacter* and *Roseburia* are bacteria that produce organic acids by fermenting carbohydrates [61,63]. In this experiment, there were no differences in relative abundances of the two bacteria between groups. It is assumed that this trend may be related to similar carbohydrate contents between corn and brown rice [15]. 

The bacterial 16S rRNA gene sequencing was used to compare the fecal microbial compositions between treatment groups. According to the alpha diversity analyses, there were no significant changes of the gut microbial evenness and richness between treatment groups at the same sampling time point. However, the PCoA plot showed the differences of the gut microbial memberships and their relative abundances between CON and GBR100 groups for finishing pigs. Based on the PCoA plot, microbial communities within groups became more similar as pigs grew. McCormack et al. [53] reported the same result as our trends that the intestinal microbiota became more homogenous among pigs over time. This is also consistent with the results reported by Guevarra et al. [64] that the alpha diversity index increases as age of pigs increases and the variability of microbiota among individual pigs decreases. Overall, the alpha and beta diversity indices indicate that ground brown rice shifted pig gut microbial communities.

Our results of the taxonomic analysis of sequence reads are consistent with the results reported by previous studies. The dominant bacterial phyla were Firmicutes and Bacteroidetes [44,53,59,61,65] and the most abundant genera were *Lactobacillus* and *Barnesiella* [44,54,56,66,67]. The Bacteroidetes produces short chain fatty acid and makes acidic environment in the gut [68], which can inhibit the growth of some intestinal pathogens such as *Escherichia coli*, *Salmonella* spp. and *Clostridium* spp. [69,70,71]. Vigor et al. [72] found that the relative abundance of *Lactobacillus* was high in pigs with high G:F. *Lactobacillus* or lactic acid bacteria makes acidic environment in the intestine and inhibit the growth of intestinal pathogens [73]. *Barnesiella* also has the ability to restrict the growth of intestinal pathogens and to limit colonization of antibiotic resistant pathogens. In addition, *Prevotella* plays an important role in the utilization of complex sugars by biodegrading and fermenting carbohydrates in the digestive system of non-ruminants [73]. However, excessive *Prevotella* may impair the establishment of more effective nutrient harvesting microbiota because of the interaction between *Prevotella* and other beneficial microbes [56].

## 5. Conclusions

Ground brown rice can be added to growing-finishing pig diets by substituting corn up to 100% without negative effects on growth performance, nutrient digestibility, and carcass characteristics of pigs and may modify gut microbiota of pigs if it is fed for a long time.

## Figures and Tables

**Table 1:** Composition of experimental diets for growing-finishing pigs (as-fed basis).

Items ^1^	Growing Period				Finishing Period			
	CON	GBR50	GBR75	GBR100	CON	GBR50	GBR75	GBR100
Ingredient (%)								
Corn	63.59	31.80	15.90	-	70.80	35.40	17.70	-
Ground brown rice	-	31.80	47.69	63.59	-	35.40	53.10	70.80
Soybean meal (44%)	32.30	32.30	32.30	32.3	25.00	25.00	25.00	25.00
Soybean oil	1.50	1.50	1.50	1.50	2.00	2.00	2.00	2.00
Limestone	0.90	0.90	0.90	0.90	1.00	1.00	1.00	1.00
Dicalcium phosphate	1.00	1.00	1.00	1.00	-	-	-	-
Iodized salt	0.20	0.20	0.20	0.20	0.20	0.20	0.20	0.20
Vitamin-Mineral premix ^2^	0.40	0.40	0.40	0.40	0.40	0.40	0.40	0.40
L-lysine-HCl	0.10	0.10	0.10	0.10	-	-	-	-
DL-methionine	0.01	0.01	0.01	0.01	-	-	-	-
Total	100.00	100.00	100.00	100.00	100.00	100.00	100.00	100.00
Analyzed energy and nutrients								
DM, %	96.36	96.47	96.62	96.38	96.72	96.41	96.56	96.52
CP, %	20.41	20.01	20.64	19.43	17.59	17.05	18.54	16.78
GE, kcal/kg	4436	4351	4446	4325	4325	4486	4275	4316
Calculated energy and nutrients								
ME, kcal/kg	3368	3438	3473	3508	3399	3477	3516	3555
CP, %	19.52	19.69	19.77	19.85	16.81	16.99	17.09	17.18
Calcium, %	0.68	0.68	0.68	0.68	0.59	0.59	0.59	0.59
Phosphorous, %	0.56	0.58	0.59	0.6	0.47	0.49	0.5	0.51
Total lysine, %	1.13	1.15	1.16	1.17	0.87	0.89	0.9	0.92
Total methionine, %	0.32	0.32	0.33	0.33	0.28	0.28	0.29	0.29
Total methionine + cysteine, %	0.66	0.66	0.65	0.65	0.58	0.58	0.58	0.57
Total threonine, %	0.75	0.76	0.76	0.77	0.64	0.65	0.65	0.66
Total tryptophan, %	0.23	0.27	0.29	0.31	0.19	0.23	0.25	0.27

^1^ CON = control diet based on corn and soybean meal; GBR50 = replacing corn with 50% of ground brown rice; GBR75 = replacing corn with 75% of ground brown rice; GBR100 = replacing corn with 100% of ground brown rice; DM = dry matter; CP = crude protein; GE = gross energy; ME = metabolizable energy. ^2^ Provided per kilogram of diet: vitamin A, 12,000 IU; vitamin D3, 2500 IU; vitamin E, 30 IU; vitamin K3, 3 mg; D-pantothenic acid, 15 mg; nicotinic acid, 40 mg; choline, 400 mg; vitamin B12, 12 μg; Fe, 90 mg from iron sulfate; Cu, 8.8 mg from copper sulfate; Zn, 100 mg from zinc oxide; Mn, 54 mg from manganese oxide; I, 0.35 mg from potassium iodide; Se, 0.30 mg from sodium selenite.

**Table 2:** Effects of replacing corn with ground brown rice on growth performance of growing-finishing pigs ^1^.

Items ^2^	CON	GBR50	GBR75	GBR100	SEM	*p*-Value
Growing period (6 weeks)						
Initial BW, kg	23.79	24.07	24.08	23.98	1.490	0.982
Final BW, kg	63.15	65.06	64.20	63.54	1.875	0.946
ADG, g/d	937	976	955	941	21.35	0.702
ADFI, g/d	2073	2116	2084	2043	78.45	0.812
G:F, g/g	0.452	0.461	0.458	0.461	0.007	0.438
Finishing period (6 weeks)						
Initial BW, kg	64.38	66.73	64.19	65.67	2.040	0.797
Final BW, kg	103.61	105.57	102.73	105.07	2.160	0.779
ADG, g/d	934	925	918	938	21.53	0.905
ADFI, g/d	3190	3168	3062	3185	96.39	0.761
G:F, g/g	0.293	0.292	0.300	0.295	0.008	0.916

^1^ Each value is the mean of 5 replicates. ^2^ CON = control diet based on corn and soybean meal; GBR50 = replacing corn with 50% of ground brown rice; GBR75 = replacing corn with 75% of ground brown rice; GBR100 = replacing corn with 100% of ground brown rice; SEM = standard error of mean; BW = body weight; ADG = average daily gain; ADFI = average daily feed intake; G:F = gain to feed ratio.

**Table 3:** Effects of replacing corn with ground brown rice on apparent total tract digestibility of growing-finishing pigs ^1^.

Items ^2^	CON	GBR50	GBR75	GBR100	SEM	*p*-Value
Growing pig						
DM, %	87.88	89.43	89.05	90.06	1.571	0.446
Energy, %	85.89	85.90	86.56	87.19	2.134	0.635
CP, %	86.44	85.77	87.63	87.19	2.179	0.274
Finishing pig						
DM, %	79.77	80.80	80.73	82.58	3.110	0.897
Energy, %	77.98	79.75	78.32	80.95	3.400	0.882
CP, %	73.47	70.98	77.78	77.31	3.940	0.519

^1^ Each value is the mean of 5 replicates. ^2^ CON = control diet based on corn and soybean meal; GBR50 = replacing corn with 50% of ground brown rice; GBR75 = replacing corn with 75% of ground brown rice; GBR100 = replacing corn with 100% of ground brown rice; SEM = standard error of mean; DM = dry matter; CP = crude protein.

**Table 4:** Effects of replacing corn with ground brown rice on carcass characteristics of finishing pigs ^1^.

Items ^2^	CON	GBR50	GBR75	GBR100	SEM	*p*-Value
Live weight, kg	111.5	114.12	114.22	114.14	1.008	0.188
HCW, kg	87.74	89.78	89.87	89.8	0.52	0.289
Dressing percentage, %	78.69	78.68	78.69	78.67	1.16	0.914
Back fat thickness, mm	20.23	21.35	19.65	21.49	0.740	0.869

^1^ Each value is the mean of 5 replicates. ^2^ CON = control diet based on corn and soybean meal; GBR50 = replacing corn with 50% of ground brown rice; GBR75 = replacing corn with 75% of ground brown rice; GBR100 = replacing corn with 100% of ground brown rice; SEM = standard error of mean; HCW = hot carcass weight.

**Table 5:** The average number of sequence reads and the alpha diversity indices of gut microbial communities in growing and finishing pigs ^1^.

Diversity Index ^2^	CON	GBR100	*p*-Value
Growing pigs			
Average no. of sequence reads per sample	181,852 ± 19,196	186,395 ± 16,116	0.769
Observed OTUs	259.00 ± 13.75	243.67 ± 3.79	0.136
Chao1	276.83 ± 18.03	267.06 ± 11.10	0.469
Shannon	4.60 ± 0.44	4.62 ± 0.54	0.954
Simpson	0.89 ± 0.03	0.89 ± 0.03	0.966
Finishing pigs			
Average no. of sequence reads per sample	217,143 ± 73,819	203,195 ± 46,636	0.796
Observed OTUs	330.33 ± 18.15	332.67 ± 17.62	0.881
Chao1	351.12 ± 23.77	351.76 ± 18.60	0.972
Shannon	4.99 ± 0.32	5.15 ± 0.26	0.524
Simpson	0.92 ± 0.01	0.93 ± 0.01	0.380

^1^ Each value is the mean of 3 replicates and is presented as mean ± standard deviation. ^2^ CON = control diet based on corn and soybean meal; GBR100 = replacing corn with 100% of ground brown rice; OTUs = operation taxonomic units.

## Data Availability

The data presented in this study are available from the corresponding author on request.
